# Supplementary material for: The implementation of remote home monitoring models during the COVID-19 pandemic in England
Source: eClinicalMedicine. 2021 Mar 30;34:100799. doi: 10.1016/j.eclinm.2021.100799 (PMC8008987; doi:10.1016/j.eclinm.2021.100799)
Supplement: Supplementary file 1 [file mmc1.docx]

***Appendix 1. Interview topic guide***

**A rapid service evaluation of remote home monitoring (‘virtual ward’) during the COVID-19 pandemic in the UK**

1. Can we start with a description of your current role?
2. How did the remote home monitoring model or pilot originate and how did it develop over time?
   1. *Why were remote home monitoring pilots needed at the time?*
   2. *Did it develop from a previous remote home monitoring model or ambulatory care pathway?*
   3. *How long did it take you to get the model up and running?*
   4. *Who led the development of the model?*
3. What were the aims of the model and its main features?
   1. *Population served*
   2. *Characteristics of patient groups*
   3. *Pre-hospital, step-down or both?*
   4. *Availability of other services at community or primary care level*
4. What were the processes involved in the pilot?
   1. *Patient triage*
   2. *Patient information and training*
   3. *Patient monitoring (what was monitored and how)*
   4. *Mechanisms used for patient data reporting (i.e. app, paper-based)*
   5. *Tools for flagging deterioration*
   6. *Escalation processes*
   7. *Patient discharge from ward*
5. What were the outcomes you expected from the implementation of the pilot? How did you think the pilot would help you achieve these outcomes?
6. What were the factors that acted as barriers and facilitators in the design and implementation of the pilots during wave 1 of the pandemic?
7. What data did you collect on patients while implementing the pilot?
8. *Have these data been linked to other data sources?*
9. *Who is able to access these data?*
10. *How have these data helped you to monitor progress against your expected outcomes?*
11. *Have there been changes to the data you’ve collected during the pilot? If yes, why were changes made?*
12. *Would you be able to share aggregated data (i.e. total number of patients, their outcomes, etc.), and lists of data fields with us?*
13. What data or information, if any, would you have liked to have collected, but couldn’t? Why?
14. What quantitative evidence, if any, did you make use of to design parts of the pilot processes and inform your decisions?
15. What resources were allocated (including staff) to implement the pilots? Do you have any documents you can share with us?
16. What was the staffing model used for the pilot?
    1. *Staff grades*
    2. *Rota*
    3. *Responsibilities*
17. What is your perception of patients’ and carers’ experiences of the pilot?
    1. *Did you have trouble accessing any patient groups?*
    2. *Did you feel any patient groups had difficulties accessing care or being onboarded onto the virtual ward?*
    3. *Do you feel patients and carers received all of the necessary information? Do you feel they understood the information?*
    4. *Were patients able to engage well with the services delivered throughout the pilot?*
    5. *Did you collect any data on patient experience? If so, are you able to share this with us?*
18. What are the lessons learnt from implementing the pilot during wave 1 of the pandemic?
    1. *Benefits of these models and areas of good practice*
    2. *Limitations of these models*
    3. *Sustainability of the models*
    4. *Areas that need to be improved*
    5. *Addressing inequalities in patient access*
19. Did you adapt the model after wave 1 of the pandemic or do you plan to adapt it now? If so, why and in what ways?
20. Would you do anything differently during wave 2?
    1. *Patient triage*
    2. *Patient information and training*
    3. *Patient monitoring*
    4. *Mechanisms used for patient data reporting (i.e. app, paper-based)*
    5. *Tools for flagging deterioration*
    6. *Escalation processes*
    7. *Patient discharge from ward*
    8. *Staffing model*
    9. *Guaranteeing patient access and addressing inequalities*
21. What advice would you give colleagues attempting to implement similar pilots in other areas of the country?
22. Is there anything else you think we should know that I have not asked you?
23. Would you be interested in participating in phase 2 of the evaluation?

***Appendix 2. RAP sheet***

| *Topic* | *Main findings* |
| --- | --- |
| *General context* |  |
| *Development of the pilot (history of why it was developed and how it changed)* |  |
| *Aims and main features of the pilot* |  |
| *Components/processes involved in pilot* |  |
| *Target patient population/s* |  |
| *Implementation stage* |  |
| *Barriers and facilitators during implementation* |  |
| *Data*    *(data collected, linkage of data, expected outcomes, use of data, who has access to data, if data has changed, data important but did not collect, able to share aggregated data)* |  |
| *Resources allocated*    *(funding, staffing, etc.)* |  |
| *Staffing model*    *(Staff grades, rota, responsibilities)* |  |
| *Perception of patients’ and carers’ experiences*    *(Trouble accessing patient groups, information for patients, patient engagement, data on patient experience)* |  |
| *Lessons learnt*    *(Benefits, limitations, sustainability areas that need to be improved)* |  |
| *Current state of pilot* |  |
| *Adapting model after wave 1* |  |
| *Things would do differently in wave 2*    *(Triage, patient information and training, monitoring, reporting, flagging deterioration, escalation, discharge, staffing model)* |  |
| *Advice to others* |  |
| *Potential evaluation questions* |  |
| *Interested in taking part in phase 2* |  |
| *Other comments/reflections* |  |
| *Number of patients served for the period April 2020 – 31 August 2020* |  |
| *The number of staff involved in setting up the pilot for the period April 2020 – 31 August 2020.* |  |
| *The resources used for setting up the pilot for the period April 2020 – 31 August 2020* |  |
| *Implementing the Pilot: The job band of the members of staff undertaking each of the activities* |  |
| *Other comments:* |  |

***Appendix 3.*** Data collection template

# Evaluation of implementation of RM pilot virtual wards for COVID-19 patients: data collection form for resources, staffing, and impact

### Patient numbers and outcomes

1. Please complete the numbers in table below.

| **Activities** | **Total number of patients during the period covered** |
| --- | --- |
| 1. Patient triaged |  |
| 1. Patient monitored |  |
| 1. Patients deteriorated and escalated |  |
| How many of the patients from the latter group (deteriorated and escalated) were advised to: |  |
| (i). Dial 111 |  |
| (ii). Dial 999 |  |
| (iii). Present to ED |  |
| How many of the deteriorated and escalated patients were: |  |
| (i). Seen in ED |  |
| (ii). Admitted to hospital |  |
| (iii). Admitted to ICU |  |
| (iv). Treated in primary care |  |
| 1. Number of deaths |  |
| 1. Number discharged from the virtual ward |  |

### Setting Up the Pilot

1. What type of staff were involved in **setting up the pilot**, and approximately how much time did they spent?

| **Staff involved in setting up the pilot** | **Staff’s band/function** | **Number of staff members involved** | **Number of total hours spent per staff** |
| --- | --- | --- | --- |
| (i). |  |  |  |
| (ii). |  |  |  |
| (iii). |  |  |  |
| (iv). |  |  |  |

1. What other resources were used in **setting up the pilot**, and approximately how much did these costs?

| **Item** | **Quantities** | **Approximate costs per unit (£)** |
| --- | --- | --- |
| Medical equipment (please list them below): |  |  |
| (i). Pulse oximeters |  |  |
| (ii). Ipads/Tablets |  |  |
| (iii). ……… |  |  |
| (iv). …….. |  |  |
| Development of patient information materials (e.g. videos) |  |  |
| (i). Video development |  |  |
| (ii). App development |  |  |
| Development of tools for flagging deterioration |  |  |
| (i). …… |  |  |
| (ii). ….. |  |  |
| Development of mechanisms for patient data reporting (i.e. app, paper-based) |  |  |
| (i). Apps |  |  |
| (ii). Paper-based |  |  |
| Other (please state) |  |  |
|  |  |  |

### Implementing the Pilot

1. What is the job band of the members of staff undertaking each of the activities below and how many hours/shifts did they spent on average per week.

| **Activities** | **Staff’s band** | **Number of staff** | **Number of hours per shift** | **Number of shifts per week** |
| --- | --- | --- | --- | --- |
| 1. Patient triage |  |  |  |  |
| 1. Patient information and training |  |  |  |  |
| 1. Patient monitoring |  |  |  |  |
| 1. Escalation processes |  |  |  |  |
| 1. Deterioration processes |  |  |  |  |
| 1. Patient discharge from virtual ward |  |  |  |  |
| 1. Management/administration |  |  |  |  |
| 1. Other (please state) |  |  |  |  |

1. Other than staff time, are there any other resources (e.g. equipment) used for each of these activities during the period covered. If so, please record this below:

| **Activities** | **Type of resources** | **Unit costs (£)** | **Quantities (total for the pilot)** |
| --- | --- | --- | --- |
| 1. Patient triage |  |  |  |
| 1. Patient information and training |  |  |  |
| 1. Patient monitoring |  |  |  |
| 1. Escalation processes |  |  |  |
| 1. Deterioration processes |  |  |  |
| 1. Patient discharge from virtual ward |  |  |  |
| 1. Management/administration |  |  |  |
| 1. Other (please state) |  |  |  |

***Appendix 4***. Staffing models

***Table 1***. Resource allocation by site

| **Sites** | **Staff’s band/function** | **Pre-hospital Model** | | **Early discharge from the hospital Model** | |
| --- | --- | --- | --- | --- | --- |
|  |  | Number of staff | Total number of hours | Number of staff | Total number of hours |
| **Total number of staff involved in setting up the pilot** | | | | | |
| Site 1 | band 5, band 8b, band 9 | 12 | 770 | - | - |
| Site 2 | - | - | - | 0 | 0 |
| Site 3 | - | 0 | 0 | - | - |
| Site 4 | GP^1^, ANP^2^, band 5 | 4 | 27 | - | - |
| Site 5 | ANP, band 5, band 7, band 9 | 6 | 46 | - | - |
| Site 6 | PA^3^ student, ST3^4^, band 9 | 3 | 240 | 1 | 58 |
| **Total number of staff involved in running the pilot** | | | | | |
| Site 1 | band 7 | 1 | 1,064 | - | - |
| Site 2 | band 5, band 8, band 9 | - | - | 4 | 2,904 |
| Site 3 | band 3, band 4, band 8a, band 8d | 22 | 13,577 | - | - |
| Site 4 | GP/ANP | 9 | 633 | - | - |
| Site 5 | ANP band 7, band 9 | 22 | 2,199 | - | - |
| Site 6 | PA student, band 6 (nurse), ED specialist, ST3, band 2 | 9 | 21,467 | 2 | 5,148 |

**Source**: Based on data provided by each site.

**Note:** 1) GP =general practitioners; 2) ANP = advanced nurse practitioners; 3) PA student =students from the faculty of Physician Associates; 4) ST3 = Specialty trainee, third year.

***Table 2.*** Staffing models by site

| **Sites** | **Setting** |
| --- | --- |
| Site 1. | Secondary care: patients monitored remotely by respiratory nurses  Primary care: delivered using federation funded primary care nurses who have worked in the community |
| Site 2. | Specialist consultant, a band 8 nurse, Outpatient Parenteral Antibiotic Therapy (OPAT) nurses who were redeployed helped with the calls (during peak also relied on help from specialist respiratory nurses). |
| Site 3. | A mix of consultants, cardiologists, five physiotherapists, three physiologists, house officers (largely data collection and completing telephone calls); medical secretaries, and a medical student setting up and monitoring the database |
| Site 4. | Small core team of GP partners, practice manager and ANP; led by senior GP partner |
| Site 5. | Delivered using three ANPs, senior and junior clinicians, and specialist registrar conducting data collection and analysis. |
| Site 6 | One ICU consultant, 1 ultrasound fellow, 1 medical student, 4 PAs, admin support and 3 furloughed middle grade ED doctors (from high-risk groups) provided assistance |
| Site 7. | Pilot lead, staff in the ED (they were able to get involved in the pathway as some of their normal work had been paused), and an ED registrar was involved in phoning patients. |
| Site 8. | Pilot lead and model delivered mainly by nursing staff based in primary care. |
